# Bioengineering Clinically Relevant Cardiomyocytes and Cardiac Tissues from Pluripotent Stem Cells

**DOI:** 10.3390/ijms22063005

**Published:** 2021-03-16

**Authors:** Emma Claire James, Eva Tomaskovic-Crook, Jeremy Micah Crook

**Affiliations:** 1 ARC Centre of Excellence for Electromaterials Science, Intelligent Polymer Research Institute, AIIM Facility, University of Wollongong, Wollongong 2500, Australia; ecj810@uowmail.edu.au; 2 Illawarra Health and Medical Research Institute, University of Wollongong, Wollongong 2500, Australia; 3 Department of Surgery, St Vincent’s Hospital, The University of Melbourne, Fitzroy 3065, Australia

**Keywords:** cardiac tissue, cardiomyocyte, human pluripotent stem cells, bioengineering, tissue modelling, regenerative therapy, electrical stimulation

## Abstract

The regenerative capacity of cardiomyocytes is insufficient to functionally recover damaged tissue, and as such, ischaemic heart disease forms the largest proportion of cardiovascular associated deaths. Human-induced pluripotent stem cells (hiPSCs) have enormous potential for developing patient specific cardiomyocytes for modelling heart disease, patient-based cardiac toxicity testing and potentially replacement therapy. However, traditional protocols for hiPSC-derived cardiomyocytes yield mixed populations of atrial, ventricular and nodal-like cells with immature cardiac properties. New insights gleaned from embryonic heart development have progressed the precise production of subtype-specific hiPSC-derived cardiomyocytes; however, their physiological immaturity severely limits their utility as model systems and their use for drug screening and cell therapy. The long-entrenched challenges in this field are being addressed by innovative bioengingeering technologies that incorporate biophysical, biochemical and more recently biomimetic electrical cues, with the latter having the potential to be used to both direct hiPSC differentiation and augment maturation and the function of derived cardiomyocytes and cardiac tissues by mimicking endogenous electric fields.

## 1. Introduction

Cardiovascular disease is the primary cause of death worldwide. In 2016, the World Health Organisation estimated that 17.9 million people died from cardiovascular disease, corresponding to 31% of deaths worldwide [1,2]. Ischaemic heart disease represents the largest proportion of cardiovascular related deaths; in 2015, approximately 8.92 million deaths globally were due to ischaemic myocardial infarctions [3]. This is particularly concerning considering that whilst cardiomyocytes are renewed in the adult heart under particular in vivo physiological or pathological conditions, their regenerative rate is limited [4,5]. More specifically, cardiomyocytes are regenerated at a rate of approximately 1% per year in healthy 25-year-old adults, progressively declining over the life span to approximately 0.45% annually in 75-year-old humans. Therefore, less than 50% of cardiomyocytes renew during an average life span [6,7]. Consequently, the rate of regeneration is insufficient to functionally restore damaged tissue via native processes necessary to overcome substantial myocardial damage and loss of cardiomyocytes associated with ischaemia [6,8].

**Figure 1:**
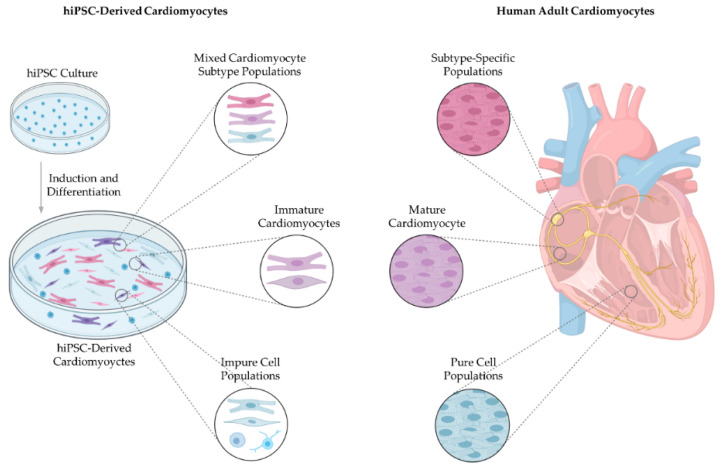
Bioengineered cardiac tissue compared to native adult cardiac tissue. Current hiPSC cardiomyocyte differentiation protocols yield mixed cardiomyocyte subtype populations with immature structure and function, and cell cultures are contaminated with non-cardiomyocytes and undifferentiated cells. Developing a hiPSC-derived cardiomyocyte differentiation process driving subtype-specific, mature and pure cell cultures will be instrumental in extending the clinical application of hiPSC-derived cardiomyocytes. hiPSCs, human-induced pluripotent stem cells.

Thus, emerging regenerative medicine therapies involving either the stimulation of endogenous regenerative processes or cell transplantation therapies have drawn increasing attention as cardiomyocyte replacement strategies [9]. Human-induced pluripotent stem cells (hiPSCs) offer much promise for developing patient-specific hiPSC-derived cardiomyocytes for modelling heart disease, pharmacology testing and regenerative replacement therapy [10,11,12] (Figure 1). However, the application of cardiomyocytes derived from hiPSCs is limited as current protocols result in mixed populations of atrial, ventricular and nodal-like cells with immature cardiac properties [13]. More specifically, hiPSC-derived cardiomyocytes display foetal-like properties in terms of morphology, electrophysiology, calcium handling, metabolism and gene expression compared to adult cardiomyocytes. Furthermore, hiPSC-derived cardiomyocyte culture methods do not provide 100% differentiation, with non-cardiomyocyte and undifferentiated cells contaminating cell hiPSC-derived cardiomyocyte cultures (Table 1) [14]. Such issues are also apparent in other leading applications for pluripotent stem cell derivatives. These include the production of oligodendrocytes, A9 dopaminergic neurons, pancreatic β-islet cells and retinal pigment epithelium to treat spinal cord injury, Parkinson’s disease, diabetes and age-related macular degeneration, respectively [15,16,17,18,19,20]. Overcoming the challenges of impurity and immaturity of iPSC-derived products is pertinent to applying hiPSC technology for regenerative medicine regardless of the disease target. Evidently, precise production of mature, subtype-specific cardiomyocytes without contaminating cells will drastically improve the translational applications of hiPSCs for disease modelling, drug screening, toxicological studies and cell therapy [21,22,23].

## 2. Clinical Need for Bioengineered Cardiomyocytes

The adult mammalian heart is not capable of repairing damaged tissue via native processes as cardiomyocytes have an insufficient regeneration rate [8]. Thus, cardiomyocytes are particularly vulnerable to multiple pathologies acute or chronic, such as apoptosis, necrosis and oncosis, which increase the risk of heart failure [24]. Dead cardiomyocytes accumulate at infarcted areas of the heart, subsequently activating host immune inflammatory responses, triggering the upregulation of cytokines and concomitant infiltration of neutrophils and macrophages to the area of necrotised tissue [25,26]. Consequently, matrix metalloproteases, a family of 25 enzymes, are activated, subsequently leading to the degradation of the surrounding coronary vasculature and the excessive extracellular matrix (ECM) turnover [27,28]. The collagen matrix becomes weakened, causing ventricular myocyte wall thinning and ventricular dilation as a result of mural realignment of myocyte bundles [29]. Fibrillary collagen deposited by fibroblast-like cells at injured sites results in the formation of scar tissue [30]. During this remodelling process, the collagen composition shifts from predominantly type I collagen to type III collagen. Hence, scar tissue with absent or reduced contraction capabilities and impulse propagation replaces regions of necrotic tissue arising from cardiomyocyte damage. The remodelling process, introducing scar tissue, becomes maladaptive due to a loss of contractile force and impulse propagation [31,32]. Hypertrophy of the remaining tissue occurs as a compensatory mechanism for the reduced cardiac function. The resultant cardiac overload is a progressive process leading to cardiac fibrosis and myocyte sliding displacement [33,34]. The end-stage of this degenerative process is heart failure, occurring when the metabolic demands of the body exceed the pumping ability of the heart. Maladaptive remodelling of the heart ultimately increases the risk of irreversible heart failure [35].

Notwithstanding significant success and progress in the treatment of myocardial infarction via pharmacological therapies, percutaneous coronary intervention or conventional coronary artery bypass graft surgery, the progression to heart failure remains high—approximately 15–30% [36,37]. Unfortunately, cardiomyocytes continue to degrade and lose contractile ability over time. Hence, patients with end-stage heart failure require more advanced treatments involving cardiac transplantation and mechanical assist devices [38]. The gold standard treatment for all causes of heart failure has been heart transplantation; however, it has a high mortality rate with a documented 15–20% mortality rate within the first year proceeding transplant [39,40]. Cardiac transplantation is also limited by the restricted availability of donor organs, potential cardiac allograft vasculopathy, malignancy and detrimental side effects associated with immunosuppression such as kidney disease, hypertension, diabetes mellitus and obesity [41]. Given the high incidence and severity of heart failure, more advanced treatments such as regenerative medicine using hiPSCs which aim to remuscularise damaged heart tissue are under investigation as potential cures for conditions associated with cardiomyocyte ischaemia [42]. Such treatments involve the implantation of exogenous hiPSC-derived cardiomyocytes to the damaged area for replacement of scar tissue with contractile tissue that is integrated electrically with the host cardiomyocytes [43].

Additionally, stem cell therapy is also being investigated as a therapeutic approach to overcome disturbances within the cardiac conduction system (CCS), particularly for pacemaker cells. The production of functional nodal cells holds great promise for advancing the treatment of cardiac disorders associated with the dysfunction or failure of sinoatrial (SA) nodal cells, collectively known as sick sinus syndrome [44,45]. The collective term sick sinus syndrome comprises sinus bradycardia, bradycardia-tachycardia syndrome, chronic atrial tachyarrhythmias, sinus pauses, sinoatrial node (SAN) exit block and inappropriate responses of heart rate to autonomic regulation [46]. Disturbances of the pacemaker cells can arise due to developmental and congenital defects, acquired injury or ischeamia or less commonly due to inherited diseases associated with alterations to the pacemaking function. The function of the SAN progressively declines with age, potentially resulting in age-related fibrosis or Lenegre–Lev syndrome [47]. Acute tissue destruction due to ischaemia or injury can occur suddenly, for example, during valve replacement surgery [48,49]. Moreover, disturbances to the cardiac conduction system may be congenital, such as congenital malformations including cardiac isomerisation and congenital heart blocks [50]. Furthermore, disturbances may arise during embryogenesis, resulting in errors in the function of the SAN and ectopic pacemakers, leading to conditions such as ventricular pre-excitation or Wolff–Parkinson–White syndrome [51]. Typically, symptomatic sick sinus syndrome requires the implantation of an electronic pacemaker [52].

There are several major drawbacks of electronic pacemakers with traditional methods of energy harvesting. For example, complications associated with technical failure of implanted systems and limited battery durability require monitoring and maintenance and increase the probability of recurrent surgery for battery and/or electrode replacement [53]. Moreover, traditional cardiac pacemakers fail to respond to the autonomic modulatory system; thereby, the heart rate fails to adapt to the demands of exercise and emotion [54]. Furthermore, the introduction of foreign material to the body is associated with infection. The incidence of pacemaker-associated infection varies from 0.1% to 20%, with 25% of pacemaker-associated infections occurring within two months of implantation. Alarmingly, cardiac pacemaker-related infection complications are associated with a mortality of up to 70%. The risk of pacemaker infection increases upon successive pacemaker related surgeries and in individuals with co-morbidities that are predisposed to pacemaker infection, such as malignancy, corticosteroid use, diabetes mellitus and skin diseases [55].

Cell-based therapies aim to address the hitherto mentioned limitations, potentially providing a long-term cure for SA nodal dysfunction. Additionally, hiPSC-derived cardiomyocytes offer opportunities to understand human heart development, cardiac disease modelling and investigation of the effects of therapeutic interventions.

## 3. Subtype-Specific Cardiomyocytes

**Figure 2:**
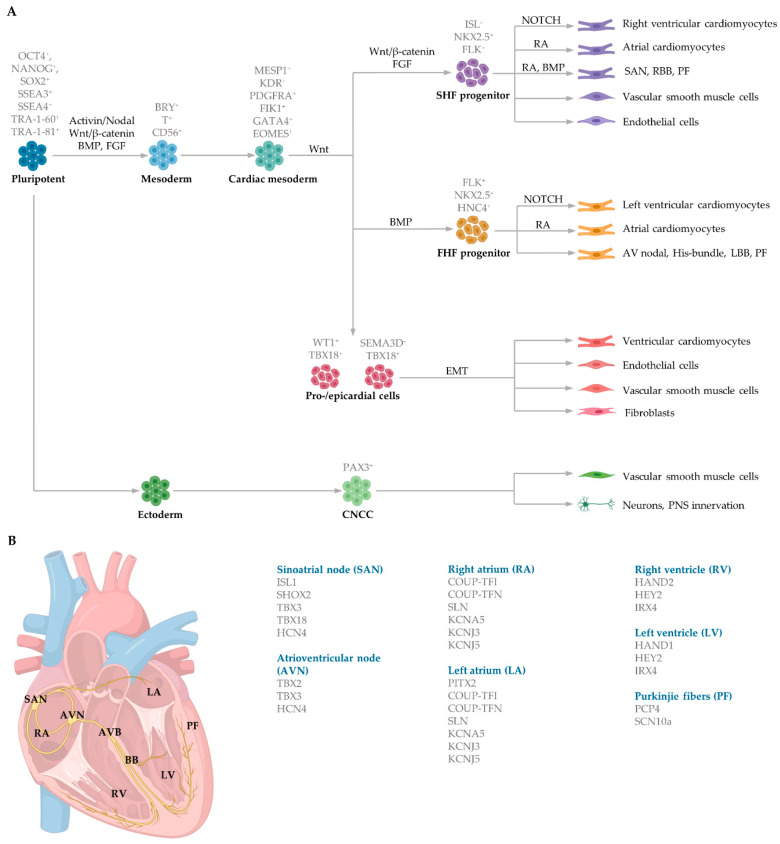
Schematic of embryonic cardiac commitment highlighting (**A**) key signalling pathways regulating each differentiation stage during cardiogenesis, including cell markers expressed during each stage, and (**B**) gene expression in distinct regions of the human heart. Mesoderm induction is induced in response to Activin/Nodal, Wnt/β-catenin, BMP, and FGF signalling. Cardiac specification is regulated by MESP1, which coordinates the migration of early cardiovascular progenitors (ISL+) to the FHF and the SHF. The FHF progenitors generate the left ventricle, portions of the atria and conducting cells. The SHF progenitors contribute to the right ventricle, portions of the atria, conducting cells, vascular smooth muscle cells and endothelial cells. Epicardial cells undergo epithelial-to-mesenchymal transition to form fibroblasts, endothelial cells, vascular smooth muscle cells and parts of the ventricle. Ectoderm progenitors also generate vascular smooth muscle and neurons responsible for autonomic innervation. EMT, epithelial-to-mesenchymal transition. CNCC, cranial neural crest cells. RA, retinoic acid.

The heart has two predominant categories of cardiomyocytes, i.e., contractile cardiomyocytes and non-contractile cardiomyocytes, which together preserve the heart’s mechanical, electrical and biochemical activity [56]. Contractile cardiomyocytes, the principal cardiomyocytes, comprise the myocardium, forming sheets of overlapping spiral patterns that attach to the fibrous skeleton of the heart [57,58]. Contractile cardiomyocytes have a distinct gene expression and cooperate with various cells of the heart, including endothelial cells, endocardial cells and epicardial-derived fibroblasts to perform the heart’s pumping action, subsequently projecting blood throughout the hearts chambers and into the peripheral circulatory system [59] (Figure 2B). The mechanical pumping is initiated by an electrophysiological stimulus to generate coordinated chronological electrical action potentials that result in muscle contraction [60].

Electrophysiological initiation and propagation are made possible by specialised non-contractile cells, the second category of cardiomyocytes present in the heart [61]. The CCS collectively refers to non-contractile cardiac cells, comprised of several nodes and conducting cells responsible for generating and transducing electrical signals to control the rate and rhythm of heart muscle contraction [62,63] (Figure 2B). These include the SAN, atrioventricular node (AVN), atrioventricular bundle, right and left bundle branches (BB) and Purkinje fibres (collectively termed the subendocardial system) [64]. Automaticity is present within cells of the SAN, AVN, atrioventricular bundle, BB and Purkinje fibres [65]. However, SA nodal cells have a relatively faster depolarisation rate compared to other components of the CCS; therefore, the pacemaker activity of the SAN overrides automaticity of other structures and sets the heart’s pace [66,67]. Although, in cases of SAN failure, the automaticity of the AVN becomes dominant, acting to secure repetitive ventricular contraction [68]. Additionally, Purkinje fibres can, to a lesser extent, act as pacemakers; this is particularly true in cases of atrioventricular block [69,70]. Gap junctions electrically connect neighbouring cardiomyocytes, allowing the transmission of electrical energy between the components of the CCS and to the rest of the heart [71]. As a result, the heart contracts as a synchronised unit independent of neural input [72]. The concomitant electrophysiological activity of the CCS and subsequent rhythmic contraction of contractile cardiomyocytes subserve the function of the heart; namely, to distribute perfused blood to the body and remove cellular waste products [73].

### 3.1. Cardiomyocyte Embryonic Specification and Differentiation

The heart is the first functional organ to form during embryogenesis [74]. Human cardiovascular system development begins during gastrulation at the end of the second week of gestation when the developing embryo’s metabolic demands exceed that supplied from placental diffusion alone [75,76]. Following gastrulation, the mammalian heart begins to develop from progenitor cells within the anterior lateral plate mesoderm [77]. On approximately day 15 of gestation, the cardiac progenitor cells within the thoracic region condense into two bilaterally located endocardial tubes on either side of the trilaminar embryo.

Mesoderm induction is regulated by several distinct and overlapping signalling pathways, including Activin and Nodal, both members of the Transforming growth factor beta (TGFβ) family, alongside the canonical (β-catenin-dependent) Wingless (Wnt) signalling pathway (Figure 2A). Bone morphogenetic proteins (BMP), also members of the TGFβ family, and fibroblast growth factors (FGF) further aid in mesoderm determination in embryos [78,79]. The transcription factors homeobox protein NKX2.5 (NK2 transcription factor related, locus 5) and mesoderm posterior basic helix-loop-helix transcription factor 1 (MESP1) play a vital role in initiating cardiac commitment. NKX2.5 collaborates with the zinc finger protein transcription factors belonging to the GATA family to induce cardiomyocyte induction. Cardiac progenitors, cardiac mesoderm cells and cardiomyocytes in the left atria and ventricle express NKX2.5 [80,81]. Mesoderm induction commences in response to the secretion of growth factors from the adjacent endoderm germ layer. These factors include BMP4 and nodal signalling. The activation of the nodal signalling pathway in the proximal region of the primitive ectoderm initiates the expression of *Bmp4* within the adjacent extraembryonic ectoderm. BMP4 in the extraembryonic ectoderm then stimulates *Wnt3* expression in the epiblast, triggering the upregulation of mesoendodermal markers such as Brachyury (Bry) and Eomesodermin (EOMES), contributing to both the definitive endoderm and the cardiac mesoderm induction [82]. EOMES-positive cells then activate MESP1 to control early cardiac progenitor commitment. MESP1 is the primary regulator of multipotent cardiac progenitor specification, regulating the induction of cardiac and mesoderm genes to activate the migration of mesodermal cells to the anterior lateral plate mesoderm via the primitive streak [79,83]. MESP1 activates Dickkopf Wnt signalling pathway inhibitor 1 (DKK1), thereby inhibiting Wnt and promoting cardiac differentiation and maturation [84,85]. Interestingly, Wnt/β-catenin signalling during cardiogenesis is biphasic. Prior to gastrulation, activation of Wnt/β-catenin signalling promotes cardiac lineage specification, whereas activation during gastrulation inhibits cardiomyocyte differentiation [86,87].

During cardiac crescent formation, two distinguishable cell populations exist: cells within the splanchnic mesoderm and cells in the epithelial structure of the crescent [77]. These different populations of progenitor cells, referred to as the first heart field (FHF) and second heart field (SHF), produce different regions of the developing heart. MESP1 controls both the distinct populations of the FHF and SHF during early gastrulation which give rise to the precursors of the subtype-specific cardiomyocytes. Early cardiac progenitors, positive for the marker ISL1, induce the production of FHF and SHF progenitor cells. FHF progenitors (ISL1- NKX2.5+), which control myocardium specification, are regulated by BMP and contribute to portions of the atria and left ventricle and the atrioventricular bundle conduction cells [88]. The SHF progenitors (ISL1 + NKX2.5+) that regulate both myocardium and endocardium specification are controlled by FGF and WNT and give rise to portions of the atria, right ventricle and SAN conduction cells [89,90] (Figure 2A).

Cardiac mesoderm progenitors coalesce, forming two paired primordia heart tubes that are composed of both endocardial and myocardial lineage. Subsequently, FHF- and SHF-derived cardiac progenitor cells proliferate within the developing structure [91]. At approximately day 21 of gestation, the bilaterally paired tubes fuse, forming a primitive linear tube. Blood flows into the tube at the caudal side and outflows at the cranial side [74]. During early heart development, dominant pacemaker activity arises at the intake region at the caudal end of the primitive tube, referred to as the sinus venosus. The embryonic cells within the sinus venosus are characterised by the expression of HCN4, encoding the potassium/sodium hyperpolarized-activated cyclic nucleotide-gated channel 4 responsible for the hyperpolarisation-activated current essential for pacemaking ability [92]. The heart tube is elongated as FHF- and SHF-derived progenitor cardiac cells rapidly proliferate and differentiate into cardiomyocytes in response to NOTCH and retinoic acid signalling from the endocardium and epicardium, respectively. Retinoic acid, the active derivative of vitamin A, modifies the expression of critical genes to induce cardiomyocyte differentiation by the induction of fibroblast growth factor signalling [93]. NOTCH coordinates cardiac specification by interaction with other signalling pathways, including WNT and BMP [94].

### 3.2. Generation of Subtype-Specific Cardiomyocytes from Human Pluripotent Stem Cells

There are a variety of protocols enabling the generation of human cardiomyocytes from iPSCs [95]. Such approaches include the use of growth factors, small molecules, genetic manipulation and biophysical cues within both monolayer and three-dimensional (3D) cultures [96]. However, these studies typically yield mixed populations of atrial, ventricular and pacemaker-like cardiomyocytes [97,98,99]. The mixed subtypes of cardiomyocytes may distort innate cellular physiology, confound disease phenotypes and jeopardize the therapeutic effects of transplanted cardiomyocytes for cardiac tissue regeneration [92]. Accordingly, at the forefront of cardiac engineering is the ability to generate subtype-specific cardiomyocytes for the precise fabrication of atrial, ventricular and pacemaker-like hiPSC-derived cardiomyocytes. Recent research has identified that subtype-specific cardiomyocytes from hiPSCs can be generated by imitating embryonic heart chamber development [100,101]. These strategies have focused on the manipulation of the critical developmental signalling pathways of cardiogenesis, in particular, WNT, NOTCH and retinoic acid signalling pathways (Table 1) [22].

NOTCH signaling is vital for ventricular cardiomyocyte specification and morphogenesis. Moreover, NOTCH regulates coronary vessel specification and is a primary modulator of arteriovenous specification during vessel development [102]. The NOTCH signalling pathway consists of two families of membrane-bound ligands, Jagged1 and 2, and Delta-like (DLL)1, 3 and 4, that bind to four transmembrane-receptors, NOTCH 1 to 4, of an adjacent cell [103]. Ligand interaction with these receptors initiates a cascade of proteolytic cleavages, including the proteolytic cleavage-by-γ-secretase leading to the NOTCH intracellular domain release from the cell-membrane and subsequent translocation to the nucleus [104]. In the nucleus, the NOTCH intracellular domain reacts with the recombining binding protein suppressor of hairless protein, resulting in the expulsion of a histone deacetylase corepressor complex, thus giving rise to an activation complex [105]. Further, Mastermind-like protein 1 and histone acetyltransferase are recruited to the site, activating the transcription of NOTCH target genes, such as the basic helix–loop–helix transcriptional repressors *HEY1* (Hairy/enhancer-of-split related with YRPW motif protein 1) and *HEY2* (Hairy/enhancer-of-split related with YRPW motif protein 2). *The activation of these NOTCH target genes results in* ventricular chamber development and spatial arrangement of cardiomyocytes within the myocardium [94].

Recent studies have demonstrated that retinoic acid signalling can promote atrial-specific differentiation of hiPSCs through the upregulation of the transcription factor COUP-TFII [106]. Conversely, the lack of retinoic acid is associated with ventricular-specific differentiation [100]. In particular, retinoic acid aids in atrial/ventricular-specific hiPSC differentiation during a restrictive temporal window proceeding mesoderm commitment [22,23]. Moreover, retinoic acid-mediated chamber specific differentiation of hiPSCs depends on the distinct mesoderm progenitor population. Different concentrations of BMP4 and Activin A induce two mesoderm populations characterised by unique cell surface markers, i.e., CD235a+ CYP26A1+ and RALDH2+ ALDH+, which generate ventricular and atrial cardiomyocytes, respectively. During hiPSC differentiation, retinoic acid signalling will efficiently give rise to atrial cardiomyocytes in RALDH2+ ALDH+ mesoderm progenitors. Comparatively, a lack of retinoic acid signalling will generate ventricular cardiomyocytes in CD235a+ CYP26A1+ progenitors [22]. Further, retinoic acid signalling combined with WNT and BMP guides the differentiation of vascular smooth muscle and cardiac fibroblast lineage from hiPSCs. Moreover, the combined treatment of RA and BMP signalling directs hiPSC differentiation towards pacemaker-like cardiomyocytes [107]. Interestingly, the inhibition of COUP-TFII signalling ablates the repressing effect on NOTCH signalling, thereby indirectly promoting ventricular differentiation [106]. Inhibition of retinoic acid signalling indirectly promotes cardiac progenitors toward a ventricular-like subtype [100]. Additionally, combined treatment with BMP4, Activin A and the WNT signalling pathway inhibitor is highly efficient at stimulating hiPSC ventricular subtype specification in differentiating cardiomyocytes [108].

The production of pacemaker-like cardiomyocytes from hiPSCs has also been achieved by mirroring embryonic pacemaker lineage specification. Sinoatrial cells originate from TBX18+ NKX2.5− mesoderm progenitors, distinguishing them from chamber-specific cardiomyocytes and other components of the CCS [109]. Pacemaker-like cardiomyocyte induction using transient stage-specific exposure to FGF and BMP signalling with concomitant inhibition of Wnt signalling produced relatively pure populations of pacemaker-like cardiomyocytes [110]. However, more recently, Ren, et al. [111] demonstrated that canonical Wnt signalling directs pacemaker-like specification of differentiating hiPSC-derived cardiomyocytes. Wnt signalling promoted enhanced expression of pacemaker-specific genes such as SHOX2, HCN4, ISL1, TBX3 and TBX18 with a concomitant reduction in NKX2.5 expression. Wnt-activated cardiomyocytes demonstrated less negative resting membrane potentials corresponding with native pacemaker electrophysiological properties, thereby functionally supporting genetic alterations. Moreover, the Wnt-activated hiPSC-derived pacemaker-like cardiomyocytes augmented pacing of bioengineered “mini heart” models. Similarly, Protze, Liu, Nussinovitch, Ohana, Backx, Gepstein and Keller [107] also used a transgene-independent method to produce pacemaker-like cardiomyocytes from hiPSCs and human embryonic stem cells by stage-specific alteration of developmental signal pathways. More specifically, FGF signalling was inhibited at the developmental stage corresponding to cardiac mesoderm induction, and BMP and retinoic acid signalling pathways were activated. Hence, the development of NKX2.5+ cardiomyocytes was eradicated, thereby directing cardiomyocyte differentiation towards a pacemaker specific lineage. Electrophysiological analysis demonstrated that the majority (90%) of pacemaker-specific cardiomyocytes had characteristic pacemaker action potentials representative of native pacemaker cardiomyocytes, suggesting typical pacemaker ion current profiles. Remarkably, when transplanted into rat hearts, the bioengineered cardiomyocytes functioned as biological pacemakers, capable of altering the pace of the host heart. Transgenic approaches to generate pacemaker-like cardiomyocytes from hiPSCs include the forced expression of SHOX2, TBX3 and TGF-beta activated kinase [112,113,114].

Alternatively, the native cellular interactions can be recapitulated in vivo to aid subtype-specific differentiation to provide an alternative protocol to achieve pacemaker-like specific cardiomyocytes. Schweizer, Darche, Ullrich, Geschwill, Greber, Rivinius, Seyler, Müller-Decker, Draguhn, Utikal, Koenen, Katus and Thomas [52] generated hiPSC pacemaker-specific cardiomyocytes by initially co-culturing hiPSCs with visceral endoderm-like (END-2) cells for ten to twelve days to form beating cell aggregates. The beating cell aggregates were then isolated and transferred to foetal bovine serum-enriched medium for further culturing for six weeks. Interestingly, the expressions of HCN1, HNC4, NCX1, Ca_v_1.2 and Ca_v_1.3 within the beating clusters were similar to, or higher than those of human sinoatrial nodal cells. Action potential measurements of cells isolated from clusters demonstrated predominantly nodal-type (63.4%) with less atrial-type (36.6%) action potential configurations and no ventricular action potential morphologies. However, the relative expression of connexin demonstrated enhanced pacemaker lineage induction, as demonstrated by a fourfold increase in Cx45 levels following differentiation. Furthermore, the beating clusters demonstrated constant and synchronised pacing of neonatal rat ventricular myocytes in co-culture experiments.

Whilst these findings aid in generating subtype-specific cardiomyocytes derived from hiPSCs, the clinical application of these methods remain limited as they yield mixed cardiomyocyte subtype-populations. Moreover, the aforementioned protocols produce cardiomyoctes with relatively immature structure and function than their native counterparts. Further refinement of the generation of subtype-specific cardiomyocytes promises to propel the translational applications of patient-derived hiPSCs for disease modelling, drug discovery/toxicity and regenerative medicine.

**Table 1:** Overview of protocols for the generation of subtype-specific cardiomyocytes involving the manipulation of cardiac development signalling pathways.

Subtype-Specific Cardiomyocyte Differentiation Protocol	Main Finding
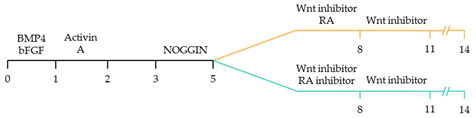	~51–65% cardiomyocyte differentiation efficiency ~94% atrial-specific cardiomyocyte differentiation ~83% ventricular-specific cardiomyocyte differentiation [100].
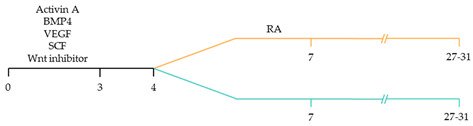	~51% cardiomyocyte differentiation efficiency ~85% atrial-specific cardiomyocyte differentiation ~80% ventricular-specific cardiomyocyte differentiation [115].
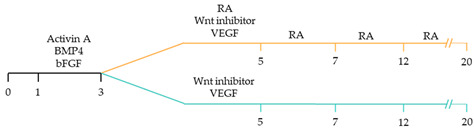	~70–98% cardiomyocyte differentiation efficiency Upregulation of atrial-specific genes following RA treatment Upregulation of ventricular-specific genes without RA treatment [22].
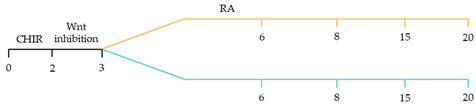	~87–90.5% cardiomyocyte differentiation efficiency ~94.7% atrial-specific cardiomyocyte differentiation ~77.8% ventricular-specific cardiomyocyte differentiation [23].
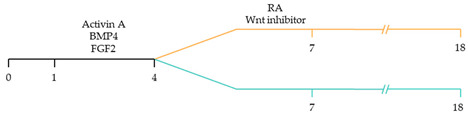	~93–94% cardiomyocyte differentiation efficiency RA treatment increased atrial specific genes, and tissue had a similar pharmacological response to muscarinic agonist as native atrial tissue [116].
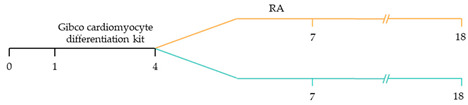	~85% atrial-specific cardiomyocyte differentiation ~86% ventricular-specific cardiomyocyte differentiation [117].
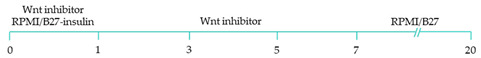	~98% cardiomyocyte differentiation efficiency ~91.5% efficiency of ventricular-specific differentiation with 87% cardiac differentiation efficiency [118].
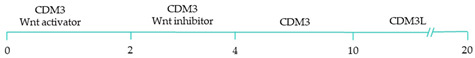	80–95% cardiac differentiation efficiency, with 50-60% of cardiomyocytes displaying ventricular specific APs and ventricular-specific genes [119].
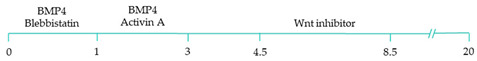	100% of cardiomyocytes displayed ventricular-like action potential parameters, with 89.42% cardiac differentiation efficiency [108].
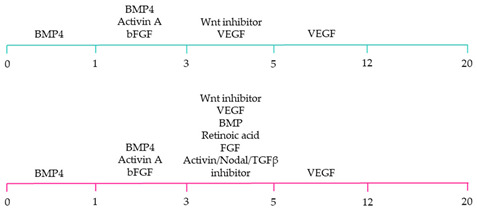	~80% cardiomyocyte differentiation efficiency. ~90% ventricular-specific differentiation ~90% pacemaker-specific differentiation. hiPSC-derived sinoatrial nodal cells demonstrated pacing ability when transplanted into rat hearts [107].
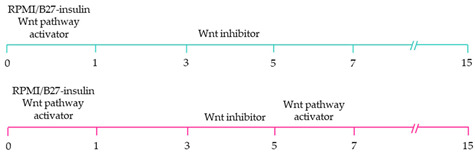	~85% cardiomyocyte differentiation efficiency. Wnt-activated hiPSC-cardiomyocytes expressed significantly elevated expression of pacemaker-related genes with pacemaker-like APs [111].
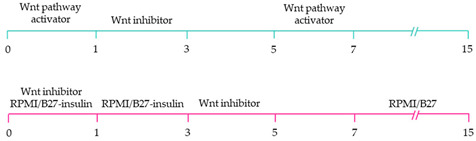	~60% cardiomyocyte differentiation efficiency. Wnt signalling promoted pacemaker lineage, whereas Wnt inhibition yielded chamber-specific cardiomyocytes [101].

Yellow, blue and pink represent atrial, ventricular and pacemaker-like cardiomyocyte differentiation, respectively. CDM3: chemically defined medium, 3 components: RPMI 1640, L-ascorbic 2-phosphate, and rice-derived recombinant human albumin. CDM3L: CDM3 without D-glucose and supplemented with sodium DL-lactate.

## 4. Maturity of Bioengineered Cardiomyocytes

Bioengineered hiPSC-derived cardiomyocytes display many of the characteristics of their native counterparts, rendering them a clinically relevant model for studying the mechanisms underlying cardiac diseases and, as a corollary, designing new pharmacological therapies. hiPSCs-derived cardiomyocytes are employed to model and trial therapeutic strategies for cardiac arrhythmias [120,121], cardiomyopathies [122,123] and metabolic disorders [124,125]. Although heart tissue was first cultured almost a century ago, most current protocols generate foetal-like cardiomyocytes that are relatively immature compared to adult cardiomyocytes, thereby limiting their clinical applications [126]. Inconsistencies with adult cardiomyocytes and the subsequent impact associated with immature characteristics (summarised in Table 2) include foetal-like morphology, poor expression of several contractile-protein and ion channel encoding genes contributing to immature electrophysiological and contractile properties. Moreover, hiPSC-derived cardiomyocytes have an adrenergic response and metabolism that are representative of foetal cardiomyocytes rather than mature adult cardiomyocytes [127]. The relative immaturity of hiPSC-derived cardiomyocytes hinders disease modelling of cardiomyopathies including metabolic syndromes, sarcomeric syndromes such as mutations of the sarcomere protein titin (TTN), disorders affecting cardiomyocyte contractility and cell cycle re-entry in mature cardiomyocytes in response to mitogens [125,128,129,130].

### 4.1. Morphology

Adult cardiomyocyte morphology provides the structural framework necessary for the execution of the functional electrophysiological and contractile properties of the cell. For example, morphological parameters including cell geometry, distribution of gap junctions and the interstitial space directly impact cell conductance [163]. In vivo cardiomyocyte hypertrophy associated with maturation following birth is linked to an alteration in the distribution of gap junctions and cell adhesion molecules. As native cardiomyocytes grow, gap junctions and cell adhesion molecules concentrate at intercalated discs located at the lateral border of cells. As such, larger, more mature cardiomyocytes have an increased maximal action potential upstroke velocity and enhanced impulse propagation velocity than smaller cardiomyocytes [164]. Current hiPSC differentiation protocols lack the biochemical and biophysical cues required to recapitulate native cardiomyocyte hypertrophy. Consequently, cultured hiPSC-derived cardiomyocytes are circular to oblong shaped, measuring 5-fold and 10-fold shorter in length and width, respectively, even after prolonged culture [132]. A reduced length-to-width ratio reduces the presence of lengthened myofibrils with laterally registered sarcomeres, thereby reducing contractility efficiency [165].

### 4.2. Electrophysiology

hiPSC-derived cardiomyocytes contract when exposed to a force of 0.08–4 mN/mm^2^, conduction velocity of approximately 10–20 cm/s and an upstroke velocity of 10–50 V/s. By contrast, in native adult cardiomyocytes, these parameters are significantly higher: 40–80 mN/mm^2^, 60 cm/s and 150–350 V/s, respectively [166]. Furthermore, hiPSC-derived cardiomyocyte cultures are typically mixed subtype populations and hence display diverse action potential phenotypes [167]. Adult atrial, ventricular and pacemaker-specific cardiomyocytes display different cellular electrophysiology due to differences in the function and expression of membrane-bound ion channels [168]. The ion channels expressed on subtype-specific cardiomyocytes have different conductances of ions, resulting in markedly different action potentials, including spontaneous diastolic depolarisation in SAN cardiomyocytes, a more negative resting membrane potential in ventricular cardiomyocytes and a less pronounced plateau in atrial cardiomyocytes.

**Figure 3:**
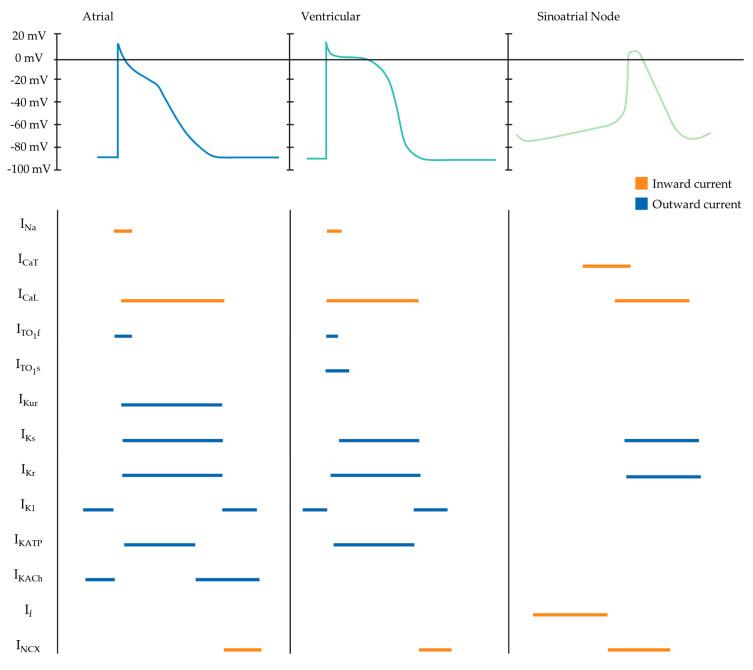
Schematic depicting atrial, ventricular and sinoatrial nodal action potential waveforms and their underlying ionic currents. Horizontal orange and blue lines represent inward and outward cellular currents, respectively. I_Na_, inward sodium. I_CaT_, T-type calcium. I_CaL_, L-type calcium. I_to1f_, fast transient outward. I_to1s_, slow transient outward. I_Kur_, ultra-rapid potassium delayed rectifier. I_Ks_, slow potassium delayed rectifier. I_Kr_, rapid potassium delayed rectifier. I_K1_, inward rectifier. I_KATP_, ADP-activated potassium channel. I_KACh_, muscarinic-gated potassium channel. I_f_, “funny” current. I_NCX_, sodium/potassium exchange current.

Contractile cardiomyocytes have a stable resting membrane proceeding depolarisation, produced by the inward rectifying potassium channel (I_K1_), whereas pacemaker cells possess intrinsic automaticity, enabled by an unstable membrane potential that begins at about −60 mV and gradually drifts towards the threshold, resulting in continuous cyclic depolarisation [168]. In pacemaker cells, hyperpolarisation proceeding an action potential opens slow sodium channels and closes potassium channels. The decrease in the conductance of the I_K1_ results in relatively lower potassium resting permeability. Once the membrane potential rises to −50 mV, the potassium/sodium hyperpolarisation-activated cyclic nucleotide-gated channel 4 (HCN4) is activated. The hyperpolarisation-activated, slow inward current of sodium, referred to as the “funny” current (If), alters the balance between potassium loss and sodium entry [169]. The sodium channel responsible for rapid depolarization (I_Na_); Nav1.5, encoded by Scn5a, is relatively low in the SAN compared to atrial and ventricular myocytes; however, SA nodal cells are characterised by a high expression of HCN4. Resultantly, the inner membrane potential is less negative, enabling slow depolarisation, therefore, reaching the threshold sooner. High expression of Nav1.5 in contractile cardiomyocytes contributes to an inward sodium current triggering rapid depolarisation. The maximum upstroke velocities for depolarisation are comparatively lower in atrial cardiomyocytes, varying between 150 and 300 V/s, compared to 300 and 400 V/s for that of ventricular cardiomyocytes [170]. The reduced upstroke velocity in atrial myocytes is attributed to the relatively depolarised resting potential compared to ventricular myocytes, thereby lowering the rapid sodium ion influx entry into the sarcolemma [171]. The succeeding plateau phase occurs as the potential approaches +30 mV, initiating concomitant voltage-gated sodium channels closure and active efflux of sodium from the cell. Simultaneously, voltage-gated L-type calcium channels (long-lasting calcium channels; I_CaL_) open, resulting in activation of ryanodine receptor type- 2 channels on the sarcoplasmic reticulum, triggering a surge in calcium concentration. The resulting entry of extracellular calcium stimulates the release of calcium from sarcoplasmic reticular stores, thereby inducing excitation–contraction coupling [172]. Compared with the spike and dome shape of the ventricular action potential, atrial action potentials exhibit a more triangular morphology with a less prominent plateau phase [173,174]. Therefore, negative voltage of the triangular atrial plateau phase increases the driving force of calcium influx, resulting in an increased inward calcium current compared to the ventricular counterpart. The I_CaL_ are solely responsible for the atrial action potential plateau phase as there is no T-type calcium (transient opening calcium channel: I_CaT_) current present [170]. The I_CaT_ forms the last portion of the pacemaker potential and is characterised by small conductances that transiently open. Atrial cardiomyocytes have a reduced inward-rectifying potassium current (approximately five- to sixfold less) compared to their ventricular counterpart, likely related to a comparatively more depolarised atrial resting membrane potential between –65 and −80 mV and the triangular, more negative plateau phase [175]. Kir2.1 is the primary molecular correlate of the I_K1_. As such, ventricular cardiomyocytes have a lower expression of Kir2.1 in comparison to atrial cardiomyocytes. Moreover, atrial cardiomyocytes possess several currents that are mostly absent in the ventricle, such as the acetylcholine-activated inward-rectifying potassium current (I_KACh_) and the ultra-rapid delayed-rectifier potassium current (I_Kur_) [176] (Figure 3).

Electrophysiological immaturity and mixed subtypes of hiPSC-derived cardiomyocytes can interfere with therapeutic applications including efficient disease modelling and drug trials. For example, hiPSC-derived cardiomyocytes often lack the inward rectifying potassium current that is critical for a stable resting membrane potential, therefore rendering them insufficient for modelling long QT syndrome [177].

Gap junction channels mediate intercellular cardiac electrical communication, allowing the transmission of coordinated electrical energy throughout the heart. Gap junctions play a critical role in the intercellular conduction velocities within the various cardiomyocyte populations; the different connexin subunit (Cx) expression among the subtype populations reflects this difference. For example, pacemaker activity within the SAN requires a high intercellular resistance (low conductivity) [66]. In the SAN, Cx45, Cx30.2 and Cx40 subunits mediate cell-to-cell coupling. These subunits form gap junctions with overall greater electrical resistance (<40 pS), which aids to shelter the SA nodal cells from the hyperpolarising current of the adjacent atrial cardiomyocytes by providing electrical isolation [178,179]. In contrast, the fast-conducting atrial and ventricular myocytes express predominantly Cx40 and Cx43 subunits that form gap junction channels of relative high conductance (low intercellular resistance) (>70 pS). The high conductance connections facilitate the rapid conduction essential for mechanical contraction [180].

Furthermore, the components of the intercalated disc complex such as N-cadherin-mediated adherens junctions, desmosomes, Nav1.5 and Cx43 polarise to the lateral ends of mature native cardiomyocytes. In vivo, the polarisation occurs gradually as the circumferentially distributed components polarise towards the long axis of the cell; however, the mechanisms mediating this process remain unknown [181]. The redistribution of these components is absent in standard cultured hiPSC-derived cardiomyocytes [182], although induced polarisation has been demonstrated by spatial restriction via micropatterned laminin surfaces [183], cyclic mechanical stretching [154] and co-culturing with bone marrow stromal cells or fibroblasts [184]. Together, it is critical to orientate the cytoarchitecture for efficient electromechanical coupling of cardiomyocytes to recapitulate native tissue and allow for the transmission of direction contraction across large distances. Cultured cardiomyocytes typically display mixed gap junction profiles, disorganized myofibrils and diffuse intercellular junctions with insufficient similarity to native adult cardiomyocytes. This further impedes the biomedical applications of hiPSC-derived cardiomyocytes.

### 4.3. Calcium Handling

Native adult cardiomyocytes develop advanced molecular machinery including T-tubules (transverse tubules) and sarcoplasmic reticulum within the sarcomere’s Z-line to modulate fast excitation–contraction coupling and calcium-induced calcium release. There are critical differences between these sophisticated structures within the various cardiomyocyte subtypes, resulting in a significantly faster atrial muscle contraction than ventricular myocyte contraction. Following calcium release from the sarcoplasmic reticulum and the initiation of contraction, reuptake of calcium into the sarcoplasmic reticulum occurs via the sarco(endo)plasmic reticulum calcium ATPase (SERCA2a), the activation of which is modulated by phospholamban (PLN) and sarcolipin (SLN). Interestingly, atrial cardiomyocytes have a higher expression of SERCA2a and lower PLN levels than ventricular cardiomyocytes [185]. However, atrial cardiomyocytes have a higher expression of SLN. Additionally, atrial cardiomyocytes also have reduced expression of ryanodine receptor type- 2 (RYR2) channels present on their sarcoplasmic reticulum and a weaker expression of the sarcoplasmic reticulum calcium buffer, calsequestrin (CASQ2), compared with that of the ventricular counterpart.

Further, ventricular myocytes express a complex system of t-tubules, consequently bringing sarcolemmal components deep to the cell interior. As a result, ventricular RYR2 channels clusters are coupled to the surface membrane via the t-tubules, resulting in a centripetal calcium wave propagation [60]. However, atrial cardiomyocytes typically lack or express only rudimentary t-tubule components. Accordingly, most atrial RYR2 channels clusters are not associated with any surface membrane contacts. Therefore, in atrial myocytes, RYR2channels closest to the sarcolemma are activated first following sequential activation of adjacent RYR2channels [186]. Together, these differences result in subtype-specific calcium handling properties of cardiomyocytes.

Consequently, in vitro cardiomyocyte cultures derived from hiPSCs typically yield mixed cardiomyocyte subtype populations, with mixed calcium handling properties consequently compromising the clinical relevance and potential therapeutic applications. Additionally, in hiPSC-derived-cardiomyocytes, sarcoplasmic reticula are foetal-like and disorganised with low expression of SERCA, and T-tubules are absent. Accordingly, hiPSC-derived cardiomyocytes depend on l-type channels for calcium release, thereby slowing fast excitation–contraction coupling. The electrophysiology immaturity further hinders the clinical relevance, in particular for disease modelling and pharmacological testing of conditions associated with dysregulated calcium handling such as catecholaminergic polymorphic ventricular tachycardia, triggered and re-entrant tachyarrhythmias and heart failure [187].

### 4.4. Metabolism

Metabolically, adult cardiomyocytes depend on fatty acids as opposed to glycolytic substrates as the preferred metabolic source. Throughout embryonic development, cardiomyocytes derive approximately 80% of energy from glycolysis following a shift to fatty acid β-oxidation during early postnatal maturation. As native cardiomyocytes mature, the mitochondrial volume increases by up to 40% and aligns between myofibrils and sarcolemma to enhance ATP production and distribution, thereby enhancing the oxidative capacity and promoting the switch in metabolic substrate [188,189,190]. Comparatively, hiPSC-derived cardiomyocytes rely predominately on glycolysis rather than fatty acid β-oxidation, although they inherently possess the capacity to utilise fatty acids [191,192].

### 4.5. Gene Expression

Classifying the genes associated with human cardiomyocyte maturation remains an enduring process. Nevertheless, the general expression patterns of maturation-related genes recognised in mice and humans are predominantly alike [193,194]. Mature cardiac tissue expresses high levels of genes such as SERCA2 (sarcoplasmic reticulum ATPase), ITPR3 (inositol-1,4,5-triphosphate), KCNH2 (potassium voltage-gated channel, CAV3 (caveolin 3) and RYR2 (ryanodine receptor 2). Additionally, indicators of cardiomyocyte maturation are also CASQ2 (calsequestrin 2), COX6A2 (cytochrome oxidase), S100A1 (S100 calcium binding protein A1), SCN5A (sodium voltage-gated channel alpha subunit 5) and MYOM2/3 (myomesin-2/3) [195]; these mature cardiac tissue markers are downregulated in hiPSC-derived cardiomyocytes [191,196,197,198,199]. Moreover, there are several discrepancies between the essential structural gene expressions of native adult cardiac tissue and hiPSC-derived cardiac tissue (summarised in Table 2). Adult cardiomyocytes display high levels of the β-isoform of the cardiac myosin heavy chain sarcomere gene (MYH7), whereas hiPSC-derived cardiomyocytes typically display elevated levels of the α-isoform (MYH6). Typically, the isoform switch occurs during the fetal to adult transition. Titin (TTN) has three predominant isoforms; N2B, N2BA and fetal cardiac titin (FCT). Adult cardiac tissue highly expresses the N2A isoform. In contrast, the N2BA isoform is predominant in hiPSC-derived cardiomyocytes. Troponin I (TnI) also has three significant isoforms including slow skeletal (ssTnI), fast skeletal (fsTnI), and cardiac (cTnI) encoded by TNNI1, TNNI2, and TNNI3, respectively. Adult cardiomyocytes display enhanced cTnI, while, the primary isoform remains ssTnI in hiPSC-derived cardiomyocytes [166,200].

Driving the maturation of hiPSC-derived cardiomyocytes is pivotal to enhance the capabilities and potential applications of hiPSC-derived cardiomyocytes. Accurate production of subtype-specific cardiomyocytes promises to progress hiPSC heart disease modelling, cardiac toxicity testing and translational medical application.

## 5. Approaches for Driving Pluripotent Stem Cell-Derived Cardiomyocyte Maturity

**Figure 4:**
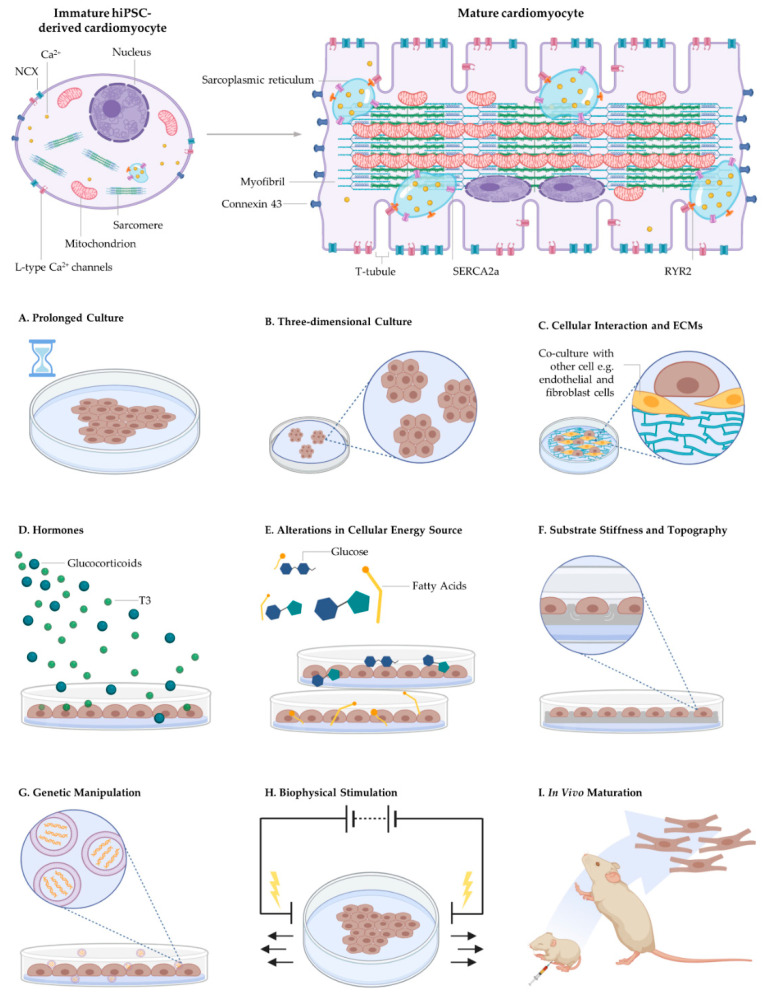
Engineered microenvironments used to augment the maturity of human-induced pluripotent stem cell-derived cardiomyocytes. Current strategies include (**A**) prolonged culture period, (**B**) three-dimensional culture periods, (**C**) co-culture with fibroblasts, endothelial cells and ECMs, and biochemical factors such as (**D**) hormones including glucocorticoids and T3 and (**E**) fatty acids rather than glucose for energy production. Approaches also include (**F**) substrate stiffness and topography, (**G**) genetic manipulation, (**H**) biophysical stimulation including mechanical and electrical stimulation and (**I**) in vivo maturation. However, the mechanisms by which these signals drive cardiomyocyte maturation remain largely unknown.

Native cardiomyocyte maturation involves the activation and integration of an orchestra of cellular signalling events. Bioengineering mature cardiomyocytes involves activating the mechanisms responsible for initiating the cellular events responsible for maturation [201]. Various approaches have been explored to accelerate maturation to overcome the immature phenotypes of hiPSC-derived cardiomyocytes such as prolonged culturing, biophysical and biochemical stimuli, genetic/epigenetic manipulation [164,202], in vivo maturation, bioengineered culture environments including 3D culturing techniques and electromechanical conditioning (Figure 4).

Prolonged cell culture was the first approach implemented to augment hiPSC-derived cardiomyocyte maturity. Long-term cell culture for forty days demonstrated augmented cellular hypertrophy, enhanced contractility, calcium handling and increased expression of ventricular-specific genes including increased CX43 expression, TnI isoform transition and MHC isoform switch [203]. Culturing for up to one year further improved sarcomeric organization; however, T-tubule development remained insufficient, indicating that prolonged cell culture alone is inadequate to mature hiPSC-derived cardiomyocytes [191]. Whilst prolonging the culture period is a simple method that facilitates maturation [135,204,205], it is neither economically nor logistically ideal, particularly in terms of up-scaling for clinical use. Further, long-term culturing is highly variable in terms of the level of maturation achieved within cultures. As such, various approaches aiming to amplify maturation within a constrained time frame are being explored.

### 5.1. Biophysical Cues

Biophysical cues such as nanogrooved substrates and micropatterned, adhesive rectangles in addition with more physiologically relevant substrate stiffness aid to recapitulate the structural features of native cardiomyocytes [131,197,206]. The use of micropatterned matrigel rectangles (2000 µm^2^ rectangles with length:width aspect ratios of 5:1–7:1) [131] and microgrooved scaffolds [197] force hiPSC-derived cardiomyocytes to adopt an elongated morphology with enhanced directionality representative of native anisotropic, rod-like shaped mature adult cardiomyocytes. The anisotropic, rod-like shape of native cardiomyocytes facilitates myofibril alignment and cell contractility. Consequently, patterning hiPSC-derived cardiomyocytes to adopt a rod-shaped morphology enhances sarcomere alignment and organisation, thereby enhancing calcium handling and subsequent force generation.

The elastic modulus of the ECM progressively increases from <10 kPa in neonatal hearts to approximately 25 kPa in adult cardiac tissue due to collagen accumulation, thereby facilitating cardiomyocyte maturation [131,207] Culturing hiPSC-derived cardiomyocytes on surfaces within physiological matrix stiffness induces enhanced sarcomere alignment, calcium handling and contractility compared with softer matrices [208,209]. Moreover, the myocardial ECM is anisotropic and fibrillar and produces essential nanotopical cues to guide cardiomyocyte alignment and assembly. These extracellular matrix features were recreated in vitro using nanofibrous scaffolds with physiologically relevant porosity to support cell infiltration and promote tissue morphogenesis, leading to enhanced native-like myocardial tissue genesis and chamber-level contractile function [210]. Decellularised myocardial matrix is an emerging strategy used to recapitulate the adult myocardial biophysical cues to guide hiPSC-derived cardiomyocyte alignment. hiPSC-derived cardiomyocytes seeded onto thin slices of decellularised porcine myocardial matrix exhibited enhanced structural and functional maturity compared to standard culture that was maintained for more than 200 days [211].

Although cardiomyocytes contribute to approximately 75% of myocardial volume, they account for only around 30% of the total cell number present in the heart. Non-cardiomyocytes such as vascular endothelial cells, cardiac fibroblasts, smooth muscle cells, immune cells and neural cells comprise the remaining portion [212]. Non-cardiomyocytes directly improve native cardiomyocyte maturation through paracrine signalling and cell-to-cell interactions [213,214,215,216]. Recreating the cardiac cell heterogeneity in vitro contributes to enhanced maturation of hiPSC-derived cardiomyocytes. Co-culturing with endothelial cells has demonstrated enhanced hypertrophy, increased expression of ion channel and sarcomere genes and an improved chronotropic response compared to monoculture hiPSC-derived cardiomyocytes [217]. Furthermore, co-culturing hiPSCs with mesenchymal stem cells improves the structural, electrophysiological and metabolic maturity of cardiomyocytes [142]. Interestingly, hiPSC-derived cardiomyocytes cultured with cardiac fibroblasts and cardiac endothelial cells within constructed, scaffold-free, three-dimensional microtissues displayed augmented electrophysiological and contractile maturity, improved sarcomeric structures with T-tubules and enhanced mitochondrial respiration compared to control tissue without multiple cardiac cell types [218].

Further research is required to establish the impact of smooth muscle cells and peripheral neurons on hiPSC-derived cardiomyocyte maturity. In summary, the aforementioned research demonstrates that the maturity of hiPSC-derived cardiomyocytes is enhanced in vitro by mirroring the native cell shape, physiological stiffness and intercellular crosstalk.

### 5.2. Biochemical Cues

Biochemical cues to improve hiPSC-cardiomyocyte differentiation and maturation include alterations in the cellular energy source to induce mainly fatty acid β-oxidation and the addition of hormones (thyroid hormone triiodothyronine (T3) and glucocorticoids) [151,219,220]. An indicator of cardiomyocyte maturation is the postnatal metabolic switch from glycolysis to fatty acid synthesis initiating hypertrophic growth and cell cycle withdrawal, thereby accelerating cardiomyocyte maturation [188,190]. The switch in adenosine triphosphate production can be mimicked in hiPSC-derived cardiomyocyte development to upregulate adult-like gene expression patterns. Supplementation of hiPSC-derived cardiomyocyte cultures with fatty acids to promote β-oxidation prevents non-cardiomyocytes induction and enriches hiPSC-derived cardiomyocytes. Substituting glucose for galactose or fatty acids, in particular, oleic acid, palmate, carnitine and linoleic acid, improved hiPSC-derived cardiomyocyte maturation. Promoting fatty acid oxidation improved morphological, structural and functional maturation as well as mitochondrial number [156,221]. Conversely, culturing hiPSC-derived cardiomyocytes with glucose enriched media promoted nucleotide biosynthesis, leading to a reduction of cardiac glucose uptake and increased nucleotide deprivation, significantly reducing cardiomyocyte maturation [222].

Alterations in metabolic hormonal signalling coincide with cardiomyocyte development and cardiovascular maturation. The serum levels of T3 surge dramatically postnatally, evoking hypertrophic growth, polyploidisation, the induction of SERCA expression and the isoform switch of myosin heavy chain and TTN [223]. The omnipresent effects of T3 are translatable to in vitro cultures for improved maturation, with T3-treated hiPSC-derived cardiomyocytes demonstrating hypertrophy, elongated morphology, increased sarcomere length, mitochondrial biogenesis and increased mitochondrial respiration, improved calcium handling and contractile properties [224]. Similarly, glucocorticoid signalling is also critical for healthy heart development, including maturation of the fetal heart. Endogenous glucocorticoid synthesis accelerates during the antepartum period, with serum levels remaining high following birth to promote cardiomyocyte maturation [225]. Glucocorticoids bind to nuclear glucocorticoid receptors (encoded by Nr3c1) expressed on cardiomyocytes to stimulate myofibril organisation and maturation [226]. Co-treatment of dexamethasone, a glucocorticoid analog, in addition with T3, further augments hiPSC-derived cardiomyocyte maturation as demonstrated by extensive T-tubule networking, improved sarcomere organisation and calcium kinetics [151]. Conversely, mutations of Nr3c1 impede cardiomyocyte maturation as indicated by sarcomere disorganisation, disruption of myocyte alignment and impaired calcium handling [145,226]. To date, research highlights the necessity of hormonal signalling and points to investigating further molecules and combinations such as angiotensin II, insulin-like growth factor and neuregulin 1β that may also have a role in cardiomyocyte maturation [227,228,229]. Together, hormone signalling and metabolic alteration can significantly enhance cardiomyocyte maturation in vitro by inducing cell cycle exit, hypertrophy, enhanced electrophysiological function and cell cycle withdrawal. Further research is required to elucidate the mechanisms behind these beneficial effects completely; however, preliminary research indicates several critical pathways such as protein synthesis, mitochondrial biogenesis and metabolic sensing and signalling.

### 5.3. Three-Dimensional Cell Culturing

Recently, 3D culture systems (with or without scaffolds) have become a physiologically relevant alternative to monolayer cultures for the maturation of hiPSC-derived cardiomyocytes. Whilst convention monolayer cultures provide simplicity and scalability, they fail to recapitulate the native extracellular microenvironment and tissue architecture, limiting cellular interaction with apical–basal polarity [230]. 3D cell cultures aim to overcome the intrinsic limitations of monolayer cell cultures by better resembling native cardiac tissue architecture [231]. In 3D cell cultures, hiPSC-derived cardiomyocytes develop improved physiological maturity than monolayer cultures, including enhanced myofibrillar organization and sarcolemma alignment, leading to enhanced calcium handling with concomitant improved contractility and electrophysiology. Importantly, hiPSC-derived cardiomyocytes mature more rapidly compared to monolayer cell cultures [96,192,232,233,234,235].

The use of 3D tissue that better resembles native cardiac tissue potentially allows for enhanced accuracy in disease modelling and drug trials to recapitulate disease phenotypes that monolayer cultures fail to represent [128,232]. While 3D cardiac tissue is a promising method for augmenting hiPSC-derived cardiomyocyte maturation, it is insufficient to produce adult-like cardiomyocytes alone. Consequently, the engineering of mature hiPSC-derived cardiomyocytes for clinical applications requires the combination of 3D cell culture with the aforementioned maturation-promoting techniques such as co-culturing [146,236,237], hormonal signalling [145,238] and electrical stimulation [136,153].

### 5.4. In Vivo Maturation

Interestingly, hiPSC-derived cardiomyocytes rapidly mature once implanted *in vivo*; however, the mechanisms responsible for the augmented maturity remain unknown [239,240]. Proceeding immediate cell death following transplantation, the in vivo environment provides the essential cues for rapid cardiac differentiation and maturation of hiPSCs. Whilst there remains dissent regarding the optimal developmental stage and health status of the host heart upon transplantation, hiPSCs mature when transplanted into a neonatal, an adult, or adult heart after infarction [239,240,241]. In vivo transplanted hiPSC-derived cardiomyocytes developed adult-like cardiomyocyte morphology, physiology and gene expression, including regular T-tubules consistent with adult calcium transients and sarcomere shortening within one month following transplantation to early postnatal rat hearts. These results indicate that hiPSC-derived cardiomyocyte maturation is accelerated within a host heart, guided by essential environmental cues, including extracellular factors [240]. In summary, in vivo studies indicate that hiPSC-derived cardiomyocytes have the capacity for extensive maturation and development of adult cardiomyocytes properties when placed into their native environment. The mechanisms governing the accelerated maturation remain unknown but are likely to include biochemical and biophysical cues, electromechanical conditioning, metabolic fuels and neurohormones in addition to potentially unknown cues.

The ability of hiPSC-derived cardiomyocytes to differentiate and mature into near-adult phenotypes following in vivo transplantation highlights that under standard cell culture conditions, critical signals from the native environment are absent. The previously mentioned methods in this section have demonstrated augmented maturity of hiPSC-derived cardiomyocytes. However, no research to date has demonstrated complete cardiomyocyte maturation in terms of structure, metabolism and electrophysiology. hiPSC-derived cardiomyocytes matured using only these methods generated cells that did not entirely mirror native adult cardiomyocytes. Hence, such approaches require the combination with further, more complex techniques. Electrical and mechanical stimulation has emerged as a potent method to augment hiPSC maturation [153,242]. Research has indicated that 3D cell culture with electromechanical conditioning and in vivo maturation via transplantation into an animal heart have tremendous success in maturing hiPSC-derived cardiomyocytes with the former presenting the more clinically amenable option [127].

## 6. Electrical Stimulation for Tissue Engineering

The overarching objective of tissue engineering and regenerative medicine is to produce tissue that is both structurally and functionally equivalent to in vivo tissue [243]. Biochemical and biophysical stimuli, including chemical mechanical, magnetic and material-based (e.g., scaffolds, topography) cues regulate native cellular activity, including cell development and maintenance (e.g., scaffolds, topography) cues [244]. Employment of these biochemical and biophysical stimuli for in vitro cell culturing has advanced tissue engineering, aiding the production of more physiologically relevant tissue [245]. More recently, electrical stimulation has been utilised to augment and influence cell behaviour in vitro. Cells and tissue within the human body are naturally bioelectric, responding to and generating electric fields for normal physiological development, function, maintenance, repair and regeneration [246]. Therein, bioelectric potentials control and regulate cell proliferation, differentiation, migration, gene expression, apoptosis and morphology. The bioelectric potentials act as long-range intercellular signals, varying depending on their physiological state, employing different signals to elicit different outcomes in cells and organs [247,248]. Thus, it is important that we understand and consider applying electrical stimuli to enhance the quality of engineered tissue that is clinically amenable for regenerative medicine [249].

Electroceuticals or bioelectronics encompasses medical devices that utilise electrical stimulation to alter and modify biological tissue. Such devices have an extensive history in human medicine with devices such as cochlear implants for auditory function, pacemakers and implantable defibrillators for cardiac arrhythmias and neuromodulation with spinal cord stimulation for chronic pain [250,251,252]. Further, the field of electroceuticals has expanded to include electrical stimulation of the vagus nerve and deep brain stimulation [253,254]. Moreover, electroceuticals may apply to a broader range of diseases, including but not limited to asthma, chronic obstructive airway, diabetes and cardiovascular and gastrointestinal diseases [255]. The use of electrical stimulation in vitro is emerging as a tool for modulating human cell behaviour for tissue engineering, modelling and regenerative medicine. The application of endogenous electrical fields to in vitro tissue is thought to better recapitulate the native environment, thereby improving control over cell proliferation, differentiation adhesion and migration [256,257,258].

### Electrical Stimulation for Bioengineering Mature Cardiac Tissue

Electrical stimulation may augment the maturity of hiPSC-derived cardiac tissue [127]. Endogenous electric fields play a critical role during normal foetal development, aiding in embryogenesis and subsequent maturation, with disturbances to these native endogenous electric fields leading to abnormal embryonic development [259]. Hence, the biomimetic paradigm has emerged, involving the application of biophysical cues, including electrical stimulation to differentiating engineered cardiac tissue to mirror the signals present in vivo to aid in producing clinically amenable bioengineered cardiomyocytes [260,261]. This led to the application of pulsatile electric fields to enhance the maturity of neonatal cardiomyocytes within 2D cell cultures. Remarkably, this preliminary work demonstrated enhanced cell alignment and sarcomere structure compared to non-stimulated controls [262]. Such findings encouraged the application of electrical stimulation of various duration, timing and frequency for enhanced differentiation of hiPSC-derived cardiomyocytes.

Interestingly, research indicates that incremental rises in stimulation intensity over the differentiation period enhance maturation compared to fixed-rate maturation. This was demonstrated within a 3D cardiac tissue platform exposed to electrical stimulation that aimed to mature hiPSC-derived cardiomyocytes [153]. hiPSCs seeded into a collagen gel surrounding a template suture in a polydimethylsiloxane microfabricated well were exposed to two different electrical stimulation protocols. The protocols involved progressive daily increments in the stimulation rate from 1 to 3 Hz (low-frequency group) and from 1 to 6 Hz (high-frequency group) [153]. Cardiac tissue subjected to electrical stimulation demonstrated augmented myofibril ultrastructural organization, additional H-zones per sarcomere, an increased number of desmosomes per membrane length and enhanced electrophysiological and calcium handling properties compatible with functional sarcoplasmic reticulum compared to non-stimulated controls. Research by Hirt, Boeddinghaus, Mitchell, Schaaf, Bornchen, Muller, Schulz, Hubner, Stenzig, Stoehr, Neuber, Eder, Luther, Hansen and Eschenhagen [136] compared two pacing protocols: 0.5 Hz for the entire stimulation period (low-frequency group) or 2 Hz in the first week and 1.5 Hz thereafter (high-frequency group). The engineered heart tissue generated from hiPSC-derived cardiomyocytes was exposed to symmetric biphasic pulses (2 ms in both polarities) of 2 V/cm via carbon electrodes. Interestingly, the high-frequency group demonstrated an augmented force of spontaneous cardiomyocyte beating. Moreover, electrically stimulated cardiac tissues had a higher cytoplasm-to-nucleus ratio with a more sophisticated muscular network composed of longitudinally oriented cardiomyocytes with an augmented contractile mass relative to the cell-free matrix in comparison to unstimulated controls. As such, these results indicate that decreasing electrical pacing frequency during cardiomyocyte differentiation enhances the structural and functional properties of hiPSC-derived cardiomyocytes for the engineering of mature cardiac tissue.

In addition to enhancing maturation, exogenous electrical stimulation can be applied to enhance cardiomyocyte subtype-specific differentiation of hiPSCs. This was initially demonstrated with human iPS(Foreskin)- 2 cells that were differentiated to cardiomyocytes by initially forming embryoid bodies (EBs) for five days [97]. The EBs were then exposed to acute biphasic current pulses at 65 mV/mm or 200 mV/mm at 1 Hz frequency and 1 ms pulse width for 5 min. The EBs were electrically stimulated in suspension via gold electrodes coated with platinum. Remarkably, electrical stimulation augmented the proportion of contracting EBs and upregulated the cardiac gene expression of ACTC1, TNNT2, MYH7, TBX5 and MYL7. Moreover, they found that electrical stimulation at 200 mV/mm for 5 min enhanced ventricular cardiomyocyte differentiation rather than atrial cardiomyocyte differentiation. Furthermore, electrically stimulated EBs showed a concentration-dependent increase in contraction frequency in response to isoproterenol, indicating increased *β*-adrenoceptor sensitivity, suggesting augmented maturity of electrically stimulated cardiomyocytes. Interestingly, the cardiogenic effect of brief electrical stimulation was not observed in a different iPSC line, CERA007c6, demonstrating that the effects of electrical stimulation vary depending on the cell line used [97].

Electrical stimulation-induced ventricular cardiomyocyte-specific differentiation was further demonstrated with EBs electrically stimulated continuously for seven days by square signals at 5 V/cm for 2 ms at 0.5, 1 or 2 Hz via carbon rods [263]. Electrically stimulated cardiomyocytes displayed augmented maturity as demonstrated by upregulated markers of cardiac differentiation (Nkx2.5), structural development (MYH6 and IGF1R), electrical development (KCNJ2) and cardiac chamber specification (NPPA, MYL2 and ANKRD1) in comparison to controls. Interestingly, electrical stimulation influenced the type of connexin expression. More specifically, the electrical stimulation enhanced the expression of GJA1 (Cx43) and CJA5 (Cx40), the predominant gap junctions present in chamber-specific cardiomyocytes and secondary ventricular cells, respectively, in comparison to controls. Moreover, the cardiomyocyte beating rate was influenced by the frequency at which they were stimulated, indicating that electrical stimulation can regulate the cardiomyocyte beating rate. The acquired beating frequency from the electrical stimulation was transferable to neighbouring cardiomyocytes and was maintained after the stimulation was discontinued.

Enhanced ventricular–cardiomyocyte differentiation was also demonstrated following electrical field conditioning of 2 Hz, beginning on day 7 post cell seeding with a weekly 1 Hz ramp-up protocol until 6 Hz was reached and maintained for one week. The stimulation frequency was then decreased to 3 Hz and maintained for up to six months [264]. The electrical field conditioning was combined with direct hiPSC-derived atrial [22] and ventricular [118] differentiation protocols. In addition to the upregulation of ventricular specific proteins such as MLC2v, electrical conditioning enhanced sarcomeric organisation, matured cellular metabolism and chamber-specific drug responses [264]. Such results indicate that electrical stimulation may enhance cardiomyocyte maturation whilst simultaneously directing differentiation towards a specific lineage. An approach that simultaneously matures and drives subtype-specific differentiation of hiPSCs-derived cardiomyocytes provides a major step towards industrial biomedical applications of engineered cardiac tissue [166].

More recent evidence highlights that electrical stimulation-augmented cardiac differentiation of hiPSCs occurs mechanistically through the activation of the calcium/PKC/ERK pathway [265]. Electric fields were produced by via a C-Pace electric stimulator consisting of two electrodes emitting bipolar stimuli of 1 or 1.5 V/cm with a biphasic square pulse (5 ms) at 5 Hz frequency for 1–30 days. Remarkably, the functional maturity of the hiPSCs was significantly augmented by electrical stimulation as demonstrated by calcium indicators, intracellular calcium levels and enhanced expression of cardiac-specific genes.

The aforementioned studies using electrical stimulation are limited by traditional tethered, penetrating wired systems, creating an inherent risk for infection and inflammation. Additionally, electrical stimulation devices may not be financially viable for industrial scalability [201]. However, the striking degree of maturity attained through combined electrical stimulation and 3D culturing techniques provides a promising approach for overcoming the immaturity of hiPSC-derived cardiomyocytes. However, the mechanisms by which electrical pacing augments cardiomyocyte maturation and induces subtype-specific differentiation are yet to be completely understood.

## 7. Discussion and Future Perspectives

Current differentiation protocols by and large yield mixed subtype populations of foetal-like cardiomyocytes, representing a significant obstacle to translational applications of hPSC-derived cardiomyocytes, including disease modelling, cell therapy and drug discovery. Whilst recently there has been much effort directed towards cardiomyocyte subtype-specific differentiation, the reliability and therefore reproducibility of methods are yet to be verified. Consistent production of ventricular, atrial and pacemaker cardiomyocytes is necessary for their application in pre-clinical and clinical testing towards translational applications. In particular, hiPSC-derived atrial-like cardiomyocytes offer a human physiologically relevant model for atrial arrhythmias and for identifying novel atrial-selective pharmacology candidates. Highly enriched ventricular cardiomyocytes would be ideal for remuscularisation of the ventricular wall to restore cardiac function following myocardial infarction. A pure ventricular cell culture, devoid of contaminating atrial and pacemaker cardiomyocytes, would help to abrogate ventricular arrhythmias observed in animal models after transplantation of grafts comprised of mixed cardiomyocyte subtypes.

Biological pacemakers have theoretical advantages compared to artificial pacemakers by providing battery independency and autonomic responsiveness. Biological pacemakers are particularly promising for infants and children with heart conditions, due to their potential adaptation during adolescence. Although effective protocols for deriving homogenous populations of atrial, ventricular- and nodal-like cardiomyocytes remain a continuing challenge, current difficulties may be circumvented through critical stage-dependent activation and inhibition of crucial development pathways and the enrichment of cardiac progenitors committed to specific subtype lineages.

Maturation of hiPSC-derived cardiomyocytes is also a major issue for the field. While specific maturation strategies (such as biochemical and biophysical cues) provide incremental advances in cardiomyocyte maturity, adult cardiomyocyte phenotypes are yet to be achieved. Collective research indicates an interdependence between specific maturation strategies with an optimal combinatory approach; in particular, including the application of electrical stimulation of 3D tissue. However, the integration of environmental cues and biophysical stimulation is yet to be optimised, and the molecular mechanisms facilitating maturation remain poorly understood.

In conclusion, future research should focus on driving cost-effective and reproducible hPSC cardiac maturation and subtype-specific differentiation, specifically through combinatorial approaches that coordinate the intensity and timing of interventions to recapitulate the native cardiac developmental process. By doing so, it is anticipated that cardiac regenerative medicine will quickly become a reality, having broad translational applications for improved therapeutics.

## Figures and Tables

**Table 2:** Different properties of foetal, hiPSC-derived and adult cardiomyocytes.

Property	Foetal Cardiomyocytes	hiPSC-Derived Cardiomyocytes	Adult Cardiomyocytes	Impact on Cell Physiology	Ref.
Morphology	Round or polygonal-shaped with chaotic organisation	Mostly round, rod-shaped cells after >50 days in culture but remain smaller than adult cardiomyocytes with chaotic organisation.	Elongated and rod-shaped with an aspect ratio of 5:1; longitudinally aligned. Larger cell size and increased membrane capacitance	Elongated structure and organised longitudinal alignment facilitate fast electrical conduction and efficient muscle contraction	[131,132]
Nucleation	Mononucleated	Largely mononucleated, showing only sporadic nucleation	25–30% binucleated	Binucleation associated with reduced regenerative potential	[132,133]
Sarcomere	1.6 μm sarcomere spacing	Irregular, 10% of cell volume, 1.6–1.8 μm sarcomere spacing	Organised and aligned, 40% of cell volume, 2.2 μm sarcomere spacing	Increased volume, organisation and spacing associated with increased force generation	[134,135,136,137]
Titin	N2BA	N2BA	N2B	Adult isoforms enhance sarcomere elasticity	[138,139,140]
Myosin heavy chain	Predominantly β-isoform in ventricular cardiomyocytes, predominantly α-isoform in atrial cardiomyocytes	Equal expression of β- and α-isoforms, potentially due to mixed cardiomyocyte sub-type cultures	β-isoform almost exclusively expressed in ventricular cardiomyocytes, α-isoform almost exclusively expressed in atrial cardiomyocytes	Adult isotype shift enhances power generation, stiffness and signalling.	[141,142]
Myosin light chain	Predominant expression of MLC2v isotype in ventricular cardiomyocytes, and predominant expression of MLC2a isotype in atrial cardiomyocytes	Equal expression of both isotypes MLC2a and MLC2v, potentially due to mixed cardiomyocyte sub-type cultures	MLC2v isotype is almost exclusively expressed in ventricular cardiomyocytes, MLC2a isotype is almost exclusively expressed in atrial cardiomyocytes	Adult isotype shift enhances power generation, stiffness and signalling	[143,144,145,146]
Metabolism	Glycolysis	Glycolysis	Oxidative phosphorylation	Fatty acid oxidation increases oxygen use and increases ATP production	[147,148]
Mitochondria structure	Small and round, close to nucleus and at periphery	Slender and long, smaller than in adult cardiomyocytes, Close to nucleus and at the periphery	Oval shape, 30% total volume, In the direction of the sarcomere	Increased density and organisation of mitochondria change substrate utilisation from glucose and lactate to fatty acids	[149,150]
T-tubule system	Poorly developed and organised	Poorly developed and organised	Highly developed and organised	A highly organised and developed T-tubule system increases synchronous and efficient calcium activation through the cell	[136,145,151,152,153]
Gap junction	Circumferential on all sides of the membrane, rather than the end	Circumferential on all sides of the membrane, rather than the end	Polarised at ends (at intercalated discs)	Increased anisotropic force generation and conduction velocity in the longitudinal direction	[136,154,155,156]
Conduction velocity	?	25–43 cm/s	70–130 cm/s	Enhanced propagation of electrical signals	[157,158]
Calcium handling	Immature calcium handling and low expression of calcium handling proteins	Immature calcium handling and low expression of calcium handling proteins	Calcium-induced calcium release is mature and efficient, high expression of calcium handling proteins	Mature calcium handling leads to fast excitation-contraction coupling, increased calcium amplitude, increased force generation, faster activation and decay and synchronised contraction in multiple sarcomeres	[135,159]
Cell cycle	Mitogens drive proliferation	Mitogens drive proliferation	Mitogens drive hypertrophy	Reduced regenerative potential and increase in cardiomyocyte size	[130,156]
Sodium ion channel expression	Foetal isoform of INa	Foetal isoform of INa	Adult isoform of INa	Faster upstroke velocity from 50 V/s in foetal and hiPSC-derived cardiomyocytes to 250 V/s in adult cardiomyocytes	[160,161]
Potassium channel expression	Low expression of IK	Low expression of IK	High expression of IK	Reduced resting membrane potential from −60 mv in foetal and hiPSC-derived cardiomyocytes to −90 mv in adults	[156]
ECM binding	β1 integrin collagen I/fibronectin	β1 integrin collagen I/fibronectin	Laminin/basement membrane	Decreased proliferation	[157,162]

INa: sodium ion channel. IK: potassium ion channel. ECM: extracellular matrix.
